# Scientific Progress in Polycystic Ovary Syndrome: Little by Little

**DOI:** 10.3390/jcm14010200

**Published:** 2025-01-01

**Authors:** Johannes Ott, Marlene Hager

**Affiliations:** Clinical Division of Gynecological Endocrinology and Reproductive Medicine, Department of Obstetrics and Gynecology, Medical University of Vienna, 1090 Vienna, Austria; marlene.hager@meduniwien.ac.at

New international recommendations on the diagnosis and treatment of polycystic ovary syndrome (PCOS) were published in autumn 2023 [1,2,3,4]. Still, the evidence for many of the recommendations is moderate to low. Moreover, concerning pathophysiology, recent Mendelian Randomization studies gave us important insights [5]. However, many details about these pathways remain unknown and many questions remain unanswered. And the more answers we find with the means of modern medical research, the more questions and hypotheses arise, forcing us to delve even deeper into the unknown.

A good example is the question of whether PCOS is associated with hyperprolactinemia, which was considered for a long time. In recent years, however, it has not only become clear that this is not the case, as reviewed by Delcour et al. [6], but also that hyperprolactinemia could have relevant positive effects on metabolism [7], which sheds a completely new light on the discussion about prolactin levels in PCOS women. Although there are a few studies about insulin resistance in women [8,9] and female patients with diabetes mellitus [10,11,12], data about PCOS patients are scarce [13,14]. However, especially in PCOS patients this issue seems intriguing. Many women affected by PCOS suffer from infertility [15] and, since hyperprolactinemia has been blamed to cause luteal phase defects [16], many clinicians tend to treat even mild hyperprolactinemia in the course of artificial reproduction. Given the possible beneficial effects of prolactin on metabolic aspects [7], such a treatment could also have negative consequences, at least hypothetically.

Another interesting point is that the idea of long-term cardiovascular risk directly caused by PCOS [1,2,3,4] has been challenged by the Mendelian randomization studies mentioned above [5]. For example, one of the most recent Mendelian randomization studies came to the conclusion that PCOS in and of itself would not increase the risk of diabetes, coronary heart disease, or stroke. It was more likely that other features of PCOS, namely obesity, elevated testosterone, and low sex hormone binding globulin (SHBG) may explain the association between PCOS and cardiometabolic diseases [17]. Notably, these aspects, especially obesity and low SHBG levels, can also be influenced by many other circumstances, one of them being lifestyle [18,19,20]. It is well-known that women with PCOS experience decreased quality of life [21,22] and are prone to suffer from more stress, depression and anxiety [23]. By providing incorrect information about long-term helöath risks, we might put even more emotional strain on the patient. Empirically, patients often search the internet to gain more information. As recently reported, PCOS-related content on websites often lack quality [24]. Considering the complexity of PCOS [25], it is difficult even for experts in gynecological endocrinology to maintain a good overview. It is therefore understandable how stressful this situation must be for patients. So, to clarify the question of long-term health consequences and what individuals really are at risk, we need long-term follow-up data with a large number of patients and many details about lifestyle, genetics, etc. There is definitely still a long way to go until then.

Given the mentioned complexity of PCOS as a metabolic and endocrine disorder, it has been hypothesized that it might derive from a mismatch between ancient genetic survival mechanisms and modern lifestyle practices, as reviewed recently [26]. Accordingly, an evolutionary model of the pathogenesis of PCOS was brought up [26]. However, given these assumptions, it is likely that also a male PCOS pattern exists. In a recent review, it is mentioned that in a “male PCOS correspondent syndrome”, genes responsible for PCOS susceptibility in women may be inherited by male relatives of women with PCOS [27]. Still, there is insufficient evidence about a “male PCOS correspondent syndrome”, although it could be of major clinical relevance concerning long-term cardiovascular risk and diabetes mellitus [27].

Another relevant topic is adolescent PCOS. According to the new international recommendation, adolescents should not be diagnosed with PCOS until they reach eight years after menarche [1]. This is based on the observation that some diagnostic criteria used to define PCOS in adult women are not applicable in adolescents, since there is an overlap with the normal physiology of a maturing reproductive system. Nonetheless, PCOS can and should be diagnosed in adolescents as well, especially young women with cutaneous signs of androgen excess [28]. It is still ammater of debate what the optimal treatment modalities would be in these patients, apart from life style interventions in adolescent girls who are overweight or obese. Many affected adolescents also experience menstrual cycle irregularities. A review published in our special issue focuses on this topic and also elaborates the use of weight-lowering treatment modalities and metformin in this population [29]. In seems relevant to mention that, given the implications of metabolic alterations in PCOS, the new Glucagon-like peptide-1 receptor agonists, will likely help overweight/obese PCOS patients by promoting weight loss and ameliorating hyperlipidemia [30]. At least, for baritric surgery, positive effects on PCOS characteristics have been published [31].

To quote the Austrian classical composer Joseph Haydn: “I listened more than I studied... therefore little by little my knowledge and ability were developed.” This also seems to apply to the field of clinical medicine: We need to listen to our patients, who tell us the needs to be met in scientific research, and to our colleagues, many of whom are willing to share their experience and knowledge. And certainly, we can all improve our knowledge and skills by doing so. In the humble opinion of the authors of this editorial, this seems to be all the more true the more complex a subject area is—and this definitely applies to the complex pattern of PCOS.

And while we all try to listen, learn and improve, we can only take small steps on this ladder. Hopefully, the articles published in this special issue on “Polycystic Ovary Syndrome: Clinical Diagnosis and Management”, published in the “Journal of Clinical Medicine”, can make a small contribution and facilitate some of the small steps on the aforementioned ladder. At least, the collection also includes studies about prolactin and cardiovascular risk in PCOS.
